# Simulation and Implementation of the Modeling of Forklift with Tricycle in Warehouse Systems for ROS

**DOI:** 10.3390/s25165206

**Published:** 2025-08-21

**Authors:** Kuo-Yang Tu, Che-Ping Hung, Hong-Yu Lin, Kaun-Yu Lin

**Affiliations:** 1 Department of Electrical Engineering, National Kaohsiung University of Science and Technology, No. 1, University Road, Yanchao District, Kaohsiung City 82445, Taiwan; 2 Ph.D. Program in Engineering Science and Technology, College of Engineering, National Kaohsiung University of Science and Technology, No. 1, University Road, Yanchao District, Kaohsiung City 82445, Taiwan4a12c062@stust.edu.tw (H.-Y.L.)

**Keywords:** robot operating system, Gmapping, A Star, time elastic band, simultaneous localization and mapping, autonomous mobile robots, tricycle forklift

## Abstract

In the age of labor shortage, increasing the throughput of warehouses is a good issue. In the recent two decades, automatic warehouses designed to reduce human labor have therefore become a very hot research topic. Tricycle forklifts being able to carry heavy goods can play important roles in automatic warehouses. Meanwhile, Robot Operating System (ROS) is a very famous and popular platform for developing the software of robotics. Its powerful communication function makes lots of warehouse information exchange easy. Therefore, ROS installed as the communication backbone of warehouse is very popular. However, the software modules of ROS do not offer tricycle forklifts. Therefore, in this research, the model of a tricycle forklift developed for ROS systems in warehouse applications is constructed. In spite of the developed model, the existing software modules must be modified for compatible connection such that the tricycle forklift can be navigated and controlled by constructed ROS. For the function of Simultaneous Localization And Mapping (SLAM) and the control of self-guided navigation, the constructed system is verified by Gazebo simulation. In addition, the experiments of a real tricycle forklift to demonstrate the developed ROS for enough accuracy of warehouse application are also included.

## 1. Introduction

Warehouses require a lot of labor to perform boring tasks. In the age of labor shortage, increasing the throughput of warehouses is therefore a good issue. In the recent two decades, automatic warehouses designed to reduce human labor have become a very hot research topic [1]. For example, Amazon Robotics [2], Boston Dynamics Stretch [3], and Daifuku’s Automated Guided Vehicle (AGV) [4] already take solutions. Amazon Robotics leads the warehouse in full autonomy. Daifuku’s AGV is designed to operate in environments with marked tracks and special markers. In contrast, the warehouse robots designed by Amazon Robotics and Boston Dynamics classified as Autonomous Mobile Robots (AMRs) are more artificial intelligence for flexible work [5]. AMRs are normally equipped with enough sensor modules and powerful computation for artificial intelligence, machine learning, and Simultaneous Localization And Mapping (SLAM), which let them navigate and operate without the specific environmental markers. Sometimes, they can also be installed by robot arms to do more tasks in warehouses, such as pick and place. AMR making use of SLAM does not need marked tracks in warehouses. Thus, the layout of warehouses installed by AMR can be changed simply and flexibly. In recent decades, AMRs have been more popular than AGV.

Traditionally, AMR is designed by two-differential drive. The two-differential drive has the limitation of payload. In large-scale warehouses such as plastic factories, many products put on a pallet result in very heavy weight. The mobile robot power of two-differential drive is not enough to carry and move the pallet. In such warehouses, the mobile robot is designed as a tricycle forklift to carry the pallet. In 2024, Alperen Keser and Pınar Oğuz Ekim studied the application of a tricycle forklift in warehouses [6]. Thus, tricycle forklifts are one of the vital modules in automatic large-scale warehouses.

Robot Operating System (ROS) is an open-source platform for robotic software development [7]. ROS offers a variety of open-source development tools that enable to the rapid creation of a robotic software system. E.N. Sardinha et al. made use of NAO and Pepper to develop a social assistant robot coaching system [8]. NAO and Pepper are very famous robot platforms based on ROS. However, they are humanoid robots. Currently, in the ROS platform for mobile robots, there are two models of drive mechanisms: the two-differential drive and the omni-directional drive. Tricycle forklifts can carry heavy goods. But there is no model for the tricycle forklifts commonly used to provide heavy payloads in big warehouses. Although the study of Alperen Keser and Pınar Oğuz Ekim for a tricycle forklift is based on ROS [6]. But it is only simulation. Therefore, the purpose of this study is to develop a software model of tricycle forklifts in ROS for the warehouse application.

To implement a navigation system for automatic warehousing with a tricycle forklift, basic technologies such as map building, global localization, global path planning [6], and trajectory following are required. These technologies are crucial for the autonomous navigation of warehouse vehicles. Common methods for map building include Gmapping [9], Hector SLAM [10], and Karto SLAM [11]. These methods involve processing data from various sensors and combining this data to construct maps. Gmapping uses laser scanners and robot motion information, Hector SLAM relies on continuous laser scans, and Karto SLAM uses both laser and odometry data. For global localization, the Monte Carlo Localization (MCL) [12,13] algorithm is commonly used to estimate the position of the warehouse vehicle. It is helpful for navigator accuracy.

In automatic warehouse vehicles, path planning is divided into global path planning and local path planning. In this study, global path planning utilizes the A* (A Star) [14,15] algorithm, which is used to calculate the path for the robot to move to its destination. When an automated warehouse vehicle is following a path determined by global path planning, the Timed Elastic Band (TEB) [16,17] trajectory following model can be incorporated. TEB is capable of adjusting the movement path to avoid obstacles by considering the motion model of the automated warehouse vehicle within a dynamic environment. This ensures that the automated warehouse vehicle can navigate around obstacles [13], enhancing the safety of the automated warehousing system.

The purpose of this study is to establish a software module for a tricycle forklift in ROS. The established software module is demonstrated in a warehouse environment constructed by Gazebo [18]. Actually, the software module is implemented by the kinematics equations of a tricycle forklift within the motion control module in ROS. This module, designed as a ROS node, can communicate with other modules in the ROS environment. The kinematics equations are the kernel to the motion controller node, which translates control commands from the navigator node into precise movements. For navigation [19], the navigator node is integrated, using a combination of the A* (A Star) algorithm and time elastic bands to efficiently plan and follow a path between two points while avoiding obstacles. The navigator node publishes control commands, which are then subscribed to by the motion controller node, where they are translated and sent to the motor controller node for execution. The entire ROS system, including the motion control and navigator modules, is demonstrated through both simulation and experiments.

The remaining sections of this paper are organized as follows. Section 2 is the Problem Description which describes the system architecture and the explored issues. Section 3 is the Research Method, where the design of the motion control module and the planning of the navigation system are introduced, combining these methods to form an unmanned warehousing system. Section 4 is the Experiment Design and Planning, which describes the experimental design. Section 5 presents the Experimental Results, showcasing the data and outcomes of the experiments. Section 6 provides the Conclusion and Future Works.

## 2. Problem Statements

**Figure 1:**
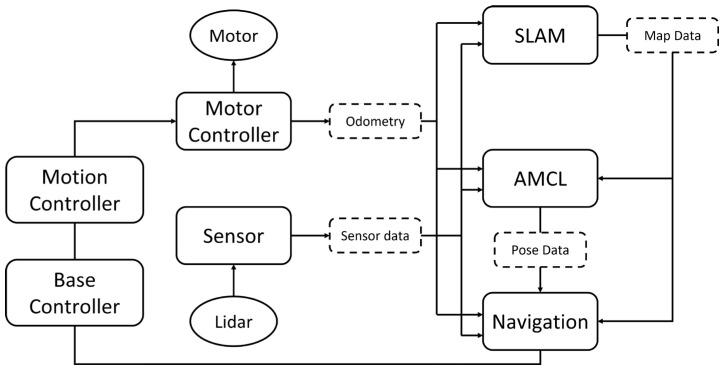
ROS-based navigation stack with four nodes—Motion Controller, Base Controller, Pose Estimation, and Path Planner—interfacing Motors, LiDAR, and Map Data to enable closed-loop path planning and execution.

In this study, the software system of ROS for vehicle navigator is designed by seven major modules: Base Controller, Motion Controller, Motor Controller, Sensor Module, Simultaneous Localization and Mapping (SLAM) [20], Augmented Monte Carlo Localization (AMCL) [21,22], and Navigation. These seven modules together constitute the software system architecture as shown in Figure 1.

As shown in Figure 1, ROS organizes robot navigator and motion control. The LiDAR sensor collects environmental data utilized for SLAM, AMCL, and Navigation modules to construct the map and to localize the robot position. The SLAM and navigation modules then interact to control the motion of the vehicle navigator. Additionally, the Navigation module processes this sensor data to plan a path and then publishes the resulting trajectory to the Base Controller. The Base Controller then forwards these commands to the Motion Controller, which converts them into motion commands in accordance with the robot’s kinematic model. Finally, these commands are published to the Motor Controller, which drives the robot along the planned trajectory.

One of the main advantages of ROS is designing nodes and topics for messages communicated between different modules. Actually, nodes are designed in modules, and topics are designed to connect nodes. Through topics, the message of nodes is flexibly communicated by subscribing and publishing. For example, the Motor Controller node communicates with the SLAM, AMCL, and Navigator nodes via ROS topics and publishes its odometry data accordingly. If the nodes of SLAM, AMCL, and Navigator nodes subscribe to the odometry topic, then they can receive the necessary data for calculating the map, localizing the robot, and planning the path, etc. Topics flexibly bridge nodes for simplifying message communication.

Generally, the vehicle kinematics in the motion controller module of ROS are based on a two-differential drive. In this paper, the control system of ROS for a tricycle forklift is studied. Therefore, we propose extending ROS with a kinematic module for a tricycle forklift to enhance overall system integration. By integrating these six modules, the system achieves fully autonomous navigation and motion control of the tricycle forklift. The first step is to establish the model and motion control system of the tricycle forklift in the ROS system, allowing the forklift to move correctly in the simulated environment. Next, sensor models will be added to the simulated tricycle forklift, enabling it to collect environmental information in the simulated environment. By combining the motion model with environmental information, SLAM and autonomous navigation can be achieved in the simulated environment.

In this paper, the major point of the research problem is to redesign Motion Controller for the motion model of a tricycle forklift. Exactly, the solution to this problem is not only to change the kinematics model of Motion Controller but also to design proper nodes for message communication. Furthermore, accommodating and validating modifications to ROS—including the addition of new nodes—presents a significant challenge within its complex software architecture that integrates diverse hardware components. Although ROS is open source, it is easy to get source code for the implementation of software systems. However, the implementation generally meets the problem of integrating the software modules developed in different compiler versions. Fortunately, ROS provides a Gazebo module for system simulation. In this paper, the implementation of the changed ROS system will be checked by the simulation. Eventually, it is implemented in a tricycle forklift for possible industrial application in the future.

## 3. Motion Control and Navigation of a Tricycle Forklift

In this paper, a ROS system for the navigation and motion control of a tricycle forklift is developed. The kinematic model of the tricycle forklift is implemented to modify the Motion Controller module in ROS as shown in Figure 1. In this section the kinematic model of a tricycle forklift is formulated to modify the Motion Controller module. Since the Navigation module in ROS also relies on the kinematic model, we include its description here to clarify the implementation.

### 3.1. Kinematic Model

**Figure 2:**
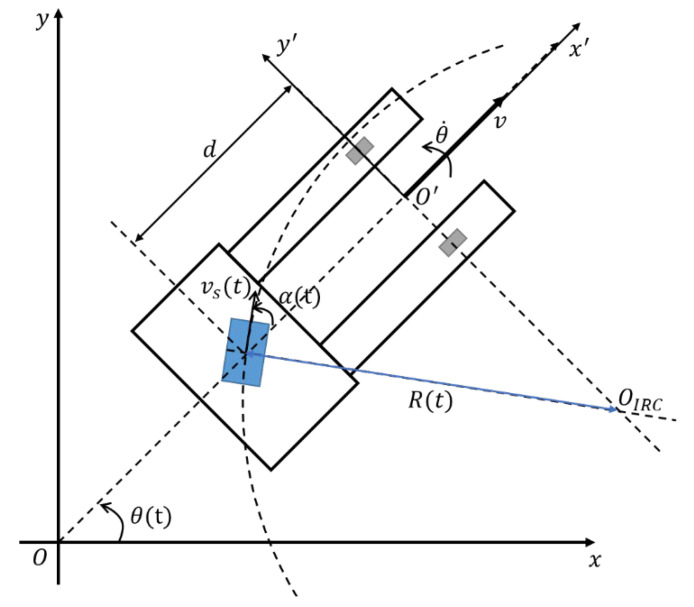
Kinematic model of a tricycle forklift showing a single steerable front wheel and two fixed rear wheels. The vehicle’s longitudinal velocity v, steering angle $\alpha \left(t\right)$, and $v_{s}\left(t\right)$ and $\omega_{s}(t)$ are linear velocity and angle velocity of steering and driving wheel.

A kinematic model of a tricycle forklift is shown in Figure 2. As shown in Figure 2, the tricycle forklift features two auxiliary front wheels and a single rear wheel responsible for both steering and propulsion. The tricycle forklift employs a three-point ground-contact configuration to provide a stable support plane for transporting heavy loads. Its single rear wheel combines steering and propulsion, enabling exceptional maneuverability in confined spaces while introducing complex kinematic behavior. As a result, tricycle forklifts have become widely adopted in warehouse operations where both stability and agility are essential.

In Figure 2, let the single rear wheel radius of the tricycle forklift be r. Then the linear and angle velocities of single rear wheel own the relationship as follows:

(1) $$v_{s}\left(t\right)=\omega_{s}(t)r$$

where $v_{s}\left(t\right)$ and $\omega_{s}(t)$ are linear velocity and angle velocity of steering and driving wheel, respectively. Let $O_{IRC}$ be the instantaneous center of tricycle forklift rotation. Then the radius of circle trajectory, R(t), keeps the following relationship

(2) $$R\left(t\right)=\frac{d}{sin\alpha (t)}$$

where α(t) is the angle of steering velocity direction in local frame, and *d* is the distance between auxiliary and single rear wheels. Notice that when $\alpha \left(t\right)=0$, R = ∞. In this situation, the move trajectory is straight line. On the circle trajectory, the velocities between linear and angle own the relationship as follows

(3) $$\omega \left(t\right)=\frac{v_{s}}{R(t)}$$

where ω(t) is the angle velocity of forklift in world frame. Substituting (2) into (3), we have

(4) $$\omega =\frac{v_{s}sin\alpha (t)}{d}$$

In general, the velocity command for navigation, u is:

(5) $$u=\left[\begin{array}{c} v\\ \omega \end{array}\right]$$

where v and ω represent the linear and angle velocities of the tricycle forklift, respectively. Note that ${\left[\begin{array}{cc} v & \omega \end{array}\right]}^{T}$ is the scale for navigation in world frame, but ${\left[\begin{array}{cc} v_{s} & \omega_{s} \end{array}\right]}^{T}$ is that for the control of the tricycle forklift in the local frame. Notice that four-wheeled forklifts can make use of this velocity directly, i.e., the two-front wheels called steeling wheels, are controlled by angle velocity ω and the two rear wheels are controlled by forward (or backward) velocity v. For differential drive, v and ω are decomposed into the velocities of the left and right wheels.

The velocity vector ${\left[\begin{array}{cc} v & \omega \end{array}\right]}^{T}$ is the command from the navigation module. The command must be transferred in exact control of the wheel for steering and driving by inverse kinematics as follows:

(6) $$v=v_{s}\left(t\right)cos\alpha \left(t\right)$$

Let (3) be divided by (5). Then we have the following:

(7) $$\frac{d\omega }{v}=\frac{sin\alpha \left(t\right)}{cos\alpha \left(t\right)}=tan\alpha \left(t\right)$$

(8) $$\alpha \left(t\right)={tan}^{-1}(\frac{d\omega }{v})$$

Note that Equation (8) provides the angle direction of a single rear wheel due to the relationship between the command velocities of linear and angle. It is a special case that $\alpha \left(t\right)=\pm \;90{}^{\circ}$. In this situation, the trajectory of the tricycle forklift is at the circle of the smallest radius d.

Let the state vector of the tricycle forklift be represented by *X* = [*x*, *y*, *θ*]^T^ in the world frame. The state vector is generally used to update the motion model in the SLAM module. The state space equations of the tricycle forklift are

(9) $$\dot{x}\left(t\right)=v_{s}\cos\alpha \left(t\right)\cos\theta \left(t\right)=\cos\theta \left(t\right)v\left(t\right)$$

(10) $$\dot{y}\left(t\right)=v_{s}\cos\alpha \left(t\right)\sin\theta \left(t\right)=\sin\theta \left(t\right)v\left(t\right)$$

(11) $$\dot{\theta }\left(t\right)=\omega (t)$$

It can be rewritten by

(12) $$\dot{X}=AX+Bu(t)$$

where A = $0_{3\times 3}$, $B=\left[\begin{array}{cc} cos\theta (t) & 0\\ sin\theta (t) & 0\\ 0 & 1 \end{array}\right]$ and $u={\left[\begin{array}{cc} v & w \end{array}\right]}^{T}$. Notice that although the kinematic Equations (9)–(11) are similar to those of four-wheeled forklifts, both have a little bit of difference. This difference can be understood by the actual driving structure of tricycles and four-wheeled forklifts. On the four-wheeled forklift, the front two wheels are controlled by steering the forklift direction, and the rear two wheels are driven to control forklift speed. However, on the tricycle forklift, the motors for steering and driving are installed on the rear wheel together. Therefore, the forward kinematics of four-wheeled and tricycle forklifts are different. For example, when the forklifts are planned by a circle trajectory, the tricycle one can be controlled to follow the circle trajectory with radius d. As shown in Figure 2, *d* is the distance between auxiliary and single rear wheels. However, the circle trajectory of a four-wheeled forklift needs a larger radius because the steering and driving wheels are installed in different locations.

### 3.2. Motion Controller Module

**Figure 3:**
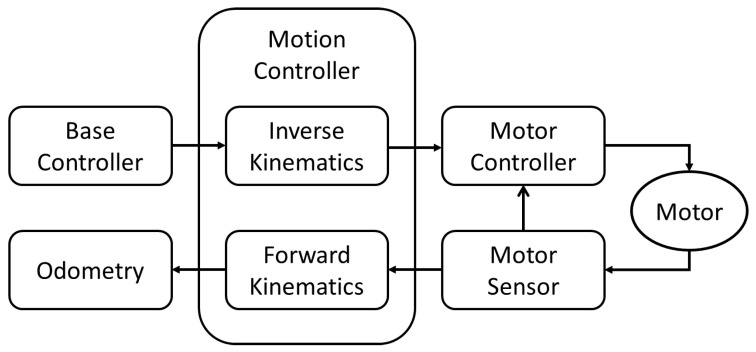
The Motion Controller uses inverse kinematics to translate Base Controller commands into Motor Controller setpoints and forward kinematics to convert Motor Sensor feedback into odometry for upstream processing.

The Motion Controller module results in the motor commands to control the tricycle forklift. The motor commands are executed by the Motor Control module to produce the speeds of steering and driving on the tricycle forklift. Meanwhile, the Motion Controller module receives the speed commands from the Base Controller, whose commands result from the trajectory planning of the Navigation module. The detailed block diagram is shown in Figure 3.

Planned by the Navigation module, the output commands from the Base Controller, $u={\left[\begin{array}{cc} v & w \end{array}\right]}^{T}$, sent to the Motion Controller are in the world frame. In the Motion Controller, the output commands must be transformed by inverse kinematics in the local frame for the commands of the Motor Controller, ${\left[\begin{array}{cc} v_{s} & w_{s} \end{array}\right]}^{T}$, as shown in Figure 3. From Equation (5), we can get the following:

(13) $$v_{s}\left(t\right)=\frac{v}{cos\alpha \left(t\right)}$$

And from Equation (1), we can get:

(14) $$\omega_{s}=\frac{v_{s}}{r}$$

In addition, we can get α(t) from Equation (8). Equations (8), (13) and (14) are the inverse kinematic Eqs. of the tricycle forklift.

As shown in Figure 3, the Motion Controller engages forward kinematics to transform the signals of the motor sensor to the posture of the tricycle forklift, [*x*, *y*, *θ*]^T^. From motor sensors, let the linear velocity of the motor be ${\hat{v}}_{s}$ and the angle direction be $\hat{\alpha }(t)$. Then the actual velocities of the tricycle forklift can be obtained from Equations (4) and (6) as follows

(15) $$\hat{\omega }=\frac{{\hat{v}}_{s}sin\hat{\alpha }(t)}{d}$$

(16) $$\hat{v}={\hat{v}}_{s}\left(t\right)cos\hat{\alpha }\left(t\right)$$

where $\hat{v}$ and $\hat{\omega }$ are the linear and angle velocities of a single rear wheel from motor sensors, respectively. Equations (15) and (16) are the forward kinematics of the tricycle forklift from motor sensors, ${\hat{v}}_{s}$ and $\hat{\alpha }(t)$. $\hat{v}$ and $\hat{\omega }$ can be engaged to derive the position and orientation of the tricycle forklift in the world frame from Equations (9)–(11). The obtained state vector [*x*, *y*, *θ*]^T^ is the output of odometry for the modules, SLAM, AMCL, and Navigation, as shown in Figure 3.

One of the ROS advantages is to communicate data between modules. It provides a node for the communication. Therefore, the ROS for the tricycle forklift can replace the nodes for inverse and forward kinematics as shown in Figure 3.

### 3.3. Navigation Module

**Figure 4:**
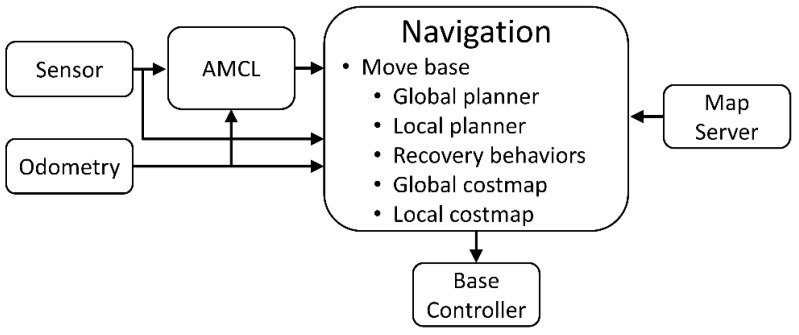
The implementation of Navigation Module.

In the ROS, the Navigation Module [16] is implemented by the block diagram as shown in Figure 4. AMCL results in the pose of a tricycle forklift for navigation. The resulting pose is calculated by the data from the models of the senor and motion based on the sensor and odometry, respectively. After getting the pose of the tricycle forklift, the navigation module makes plans according to the map from the Map Server. To integrate all works in the Navigation module, ROS provides a transformation system [23] for coordinate transformation. Finally, the Navigation module produces the control command of the tricycle forklift to the base controller.

The Navigation module makes use of the navigation stack model to get the control command sent to the Base Controller. In the navigation stack model, based on the pose of the tricycle forklift, produced by AMCL and Gmapping in Map Server, path planning decides the goal position in the global coordinate system. The goal position can be used to calculate the control command by the kinematics of the tricycle forklift.

**Figure 5:**
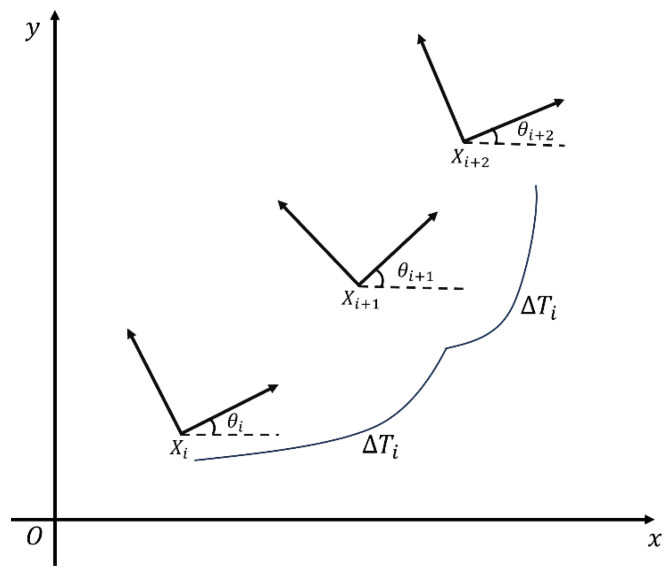
TEB optimizes a sequence of time-stamped poses by jointly adjusting spatial waypoints and time intervals to minimize path length, traversal time, and turning effort. Velocity/acceleration limits and obstacle-avoidance costs are enforced to ensure a smooth, feasible local trajectory.

Path planning can be broadly categorized into two types. The global planner computes an optimal route over a fully known map; in this approach, the A* algorithm is typically used to find the shortest obstacle-free path between a start and a goal. The local planner then refines that trajectory in real time by adapting the vehicle’s immediate course to avoid unexpected or dynamic obstacles while following the global plan. The local planner typically employs the Timed Elastic Band (TEB) algorithm, integrating real-time data from LiDAR, wheel odometry, and other sensors to adjust the trajectory dynamically and avoid obstacles. The TEB method involves adding n control points along the path and inserting a time interval T between the points. This creates point-to-point segments with time intervals T, which are then used to optimize and constrain the path as shown in Figure 5.

In the TEB method, let $X_{i}$ (for i = 1, 2, …, n) be the robot pose ${\left[x_{i},y_{i},\theta_{i}\right]}^{T}$, and define the queue in the planned trajectory of the robot pose be:

(17) $$v_{s}\left(t\right)=\frac{v}{cos\alpha \left(t\right)}$$

In the TEB, a time interval ${\Delta T}_{i}$ is inserted between $T_{i}$ and $T_{i+1}$, which are the times of robot poses at $T_{i}$ and $T_{i+1}$, respectively. Let the n−1 time intervals be defined as a sequence as follows:

(18) $$\tau ={\left\{\Delta T_{i}\right\}}_{i=\;1,2,\;\ldots ,\;n-1}$$

TEB is composed of the robot poses on the planned trajectory defined by the set as follows:

(19) $$B:=\left(Q,\tau \right)=\{X_{1},\;\Delta T_{1},\;X_{2},\;\Delta T_{2},\;\ldots ,\;\Delta T_{n-1},\;X_{n}\}$$

The major objective of the TEB algorithm is to adjust the local trajectory path for better tracking performance by using a weighting function. Let the TEB algorithm be defined as

(20) $$f\left(B\right)=\sum_{k=0}^{n-1}\gamma_{k}f_{k}\left(B\right)$$

where $f_{k}\left(B\right)$ is the constraint function in its time interval, and is the weight corresponding to the constraint function. Then the optimal path can be searched by

(21) $$B^{*}=\underset{B}{\mathrm{argmin}}f\left(B\right)$$

**Figure 6:**
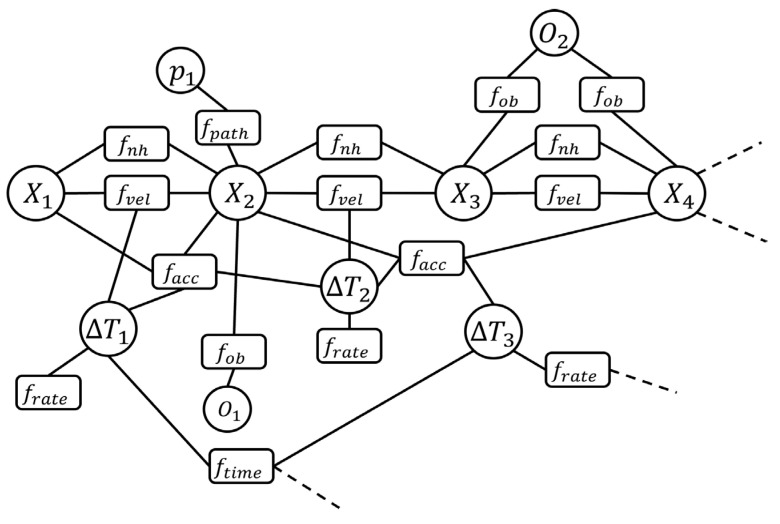
A Hyper-Graph example of TEB algorithm. This example is used to illustrate the robot poses from *X*_1_ to *X*_4_.

The TEB algorithm is implemented by Equations (20) and (21), which allow the robot to follow the global trajectory path while achieving functionalities such as obstacle avoidance. In TEB, the weighting functions are divided into two basic types. The first is a penalty function such as the limitation of velocity and acceleration. The second is an objective function for the shortest path. According to Equation (21), the TEB algorithm is defined as a quantified multi-objective optimization problem. Therefore, it is represented using a Hyper-Graph [24], which expresses the relationship between two adjacent states as shown in Figure 6.

To describe the TEB algorithm in detail, Figure 6 is an example of Hyper-Graph. This example is engaged to illustrate the structure of the TEB and the problem of multi-objective optimization, which includes the constrained states and objective functions of robot poses from $X_{1}$ to $X_{4}$ in sequence *Q*. Taking $X_{1}$ to $X_{2}$ as an example, the speed objective function $f_{vel}$ constrains the interval time between the two states with ${∆T}_{1}$, limiting the moving speed on the robot’s trajectory path. This means the robot needs to move from state $X_{1}$ to $X_{2}$ within the time ${∆T}_{1}$. $f_{acc}$ is the acceleration limit for the robot moving to $X_{2}$. $f_{ob}$ represents a minimum distance constraint between obstacle $O_{1}$ and state $X_{2}$. $f_{nh}$ provides non-holonomic constraints during the robot’s movement, and $f_{path}$ is the constraint function for the shortest path $p_{1}$ given by the TEB algorithm from pose $X_{1}$ to $X_{2}$.

The TEB algorithm takes into account the geometric and kinematic constraints of the robot model, as well as incorporating relevant dynamic constraints during the robot’s movement. This allows it to produce a local path to follow the global path trajectory, enhancing the robot’s flexibility and accuracy during navigation and movement.

## 4. Experimental Design

In this study, a tricycle forklift module is established in the ROS system. The system performance will be verified by experiments. Although a forklift with a tricycle is not as stable as four-wheels, its higher relative flexibility and smaller size lead to lower overall costs. Therefore, tricycle forklifts are still widely used in various fields. This research conducts the tests of both simulation and actual platform.

**Figure 7:**
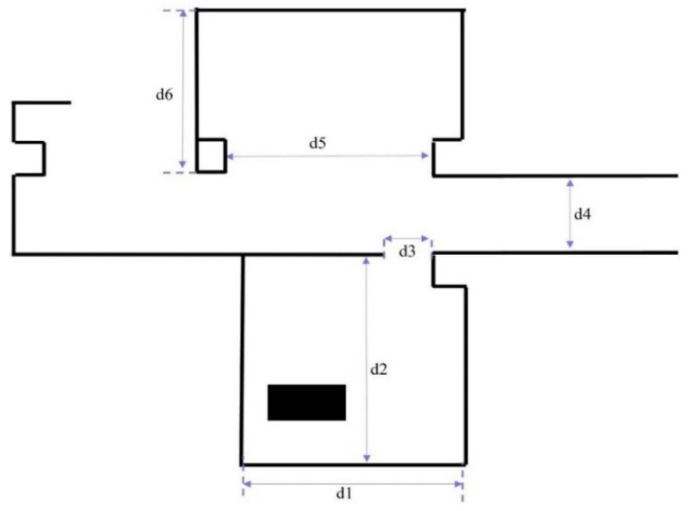
Floor-plan layout of the experimental environment, illustrating a classroom area connected to adjacent corridors.

In this study, the classrooms and their surroundings at our institution are designed as the experimental environment. The spatial parameters of the classroom as shown in Figure 7 are listed by length in Table 1.

**Figure 8:**
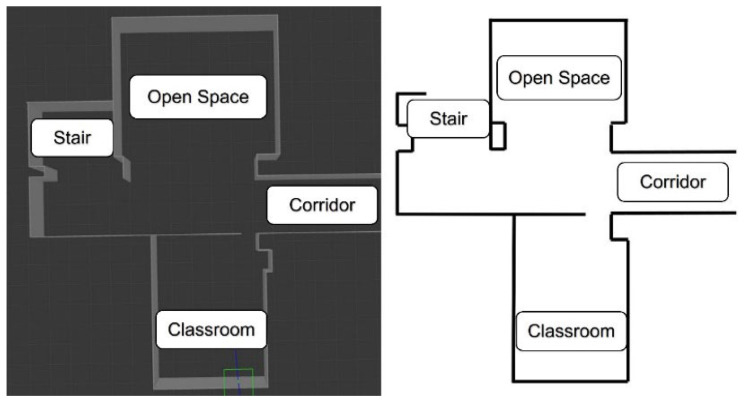
(**Left**) experimental environment simulated in Gazebo for planning the path of the tricycle forklift; (**right**) the actual experimental environment.

In this study, the Gazebo simulator of ROS is used to create simulation environments for testing. Gazebo can be developed to simulate various physical phenomena of the real-world environment in three dimensions based on the construction and application of custom-developed models. Figure 8 shows the spatial arrangement in both the actual environment (right) and the Gazebo simulation environment (left).

**Figure 9:**
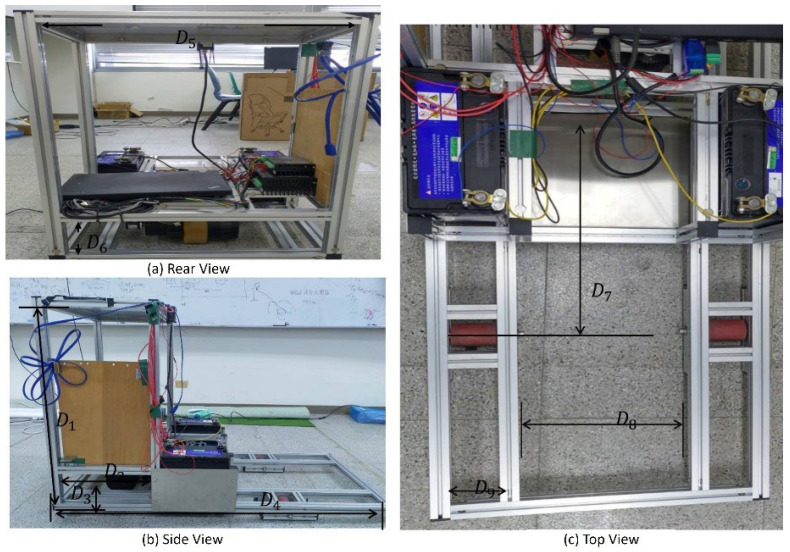
A scale model of the tricycle forklift, fabricated from aluminum extrusions, for experiments in a confined test area.

In this study, a real tricycle forklift as shown in Figure 9 is developed for demonstrating the modified ROS system. It is a scaled-down version of an actual tricycle forklift generally applied in industrial storage. Figure 9a–c represents the rear view, side view, and top view, respectively. The parameters of the tricycle forklift are detailed in Table 2.

## 5. Experiment Results

To control and navigate the tricycle forklift, most of the ROS modules are implemented by an Industrial Personal Computer (IPC). Actually, the IPC is designed to include the modules of SLAM, AMCL, Navigation, Base Controller, and Motion Controller. Then the IPC receives the sensor signal of LiDAR for SLAM, AMCL, and Navigation. After having a global path with the Navigation module, the Motion Controller module results in a command sent to control the motor of the tricycle forklift.

The experiments with the tricycle forklift involve localization and global navigation planning within an environment using a pre-built map. At the outset of the experiments, data from a 360° LiDAR scanner and motor encoders were used to generate an initial map of the environment via the Gmapping algorithm. During subsequent operation, LiDAR measurements served as the sensor model, and encoder readings provided the motion model for pose estimation using the AMCL.

**Figure 10:**
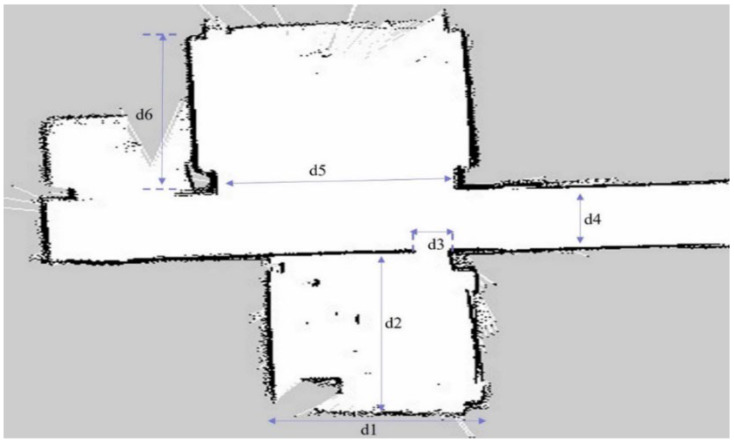
Map of the experimental environment generated by the tricycle forklift using Gmapping.

In this experiment, classrooms and corridor spaces of our school are designed as the experimental environment. The environment map constructed by Gmapping is shown in Figure 10. The comparison errors between the constructed environment map and the actual environment are presented in Table 3.

**Figure 11:**
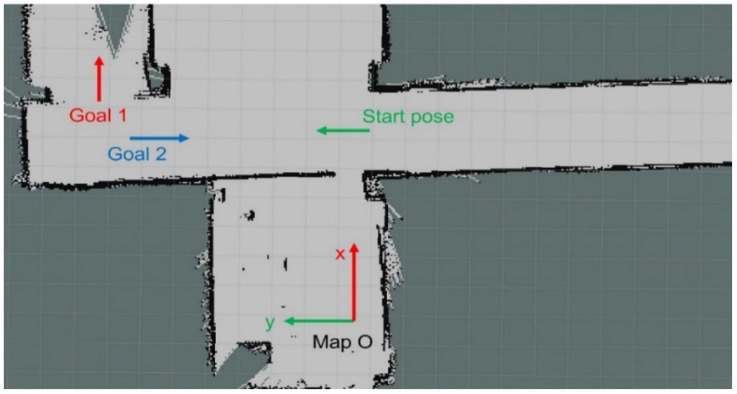
“Map O” denotes the map’s coordinate origin. The experiment plans paths from the start pose to Goal 1 and Goal 2 and records the tricycle forklift’s actual motion data.

In Figure 11, “Map O” is the grid map original point. “Start pose” is the initial pose of the tricycle forklift in the map coordinates. “Goal 1” is the first target pose, and “Goal 2” is the second target pose. The experiment is divided into two phases of movement. In the first phase, the tricycle forklift moves from the Start pose to the first target pose, Goal 1, by the full autonomous navigation developed in the ROS system. In the second phase, it turns back to move from Goal 1 to Goal 2. The robot poses on the global trajectory are recorded at every moment during both phases of the journey. This allows for the observation of the trajectory tracking capability of the tricycle forklift and to compare the error between the expected pose upon reaching each target pose and the actual pose.

**Figure 12:**
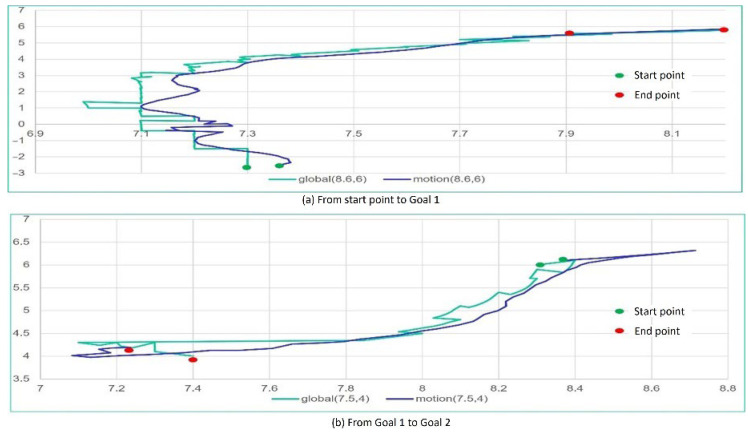
The position trajectory of the tricycle forklift with autonomous navigation in Gazebo. (**a**) is the trajectory from start pose to Goal 1, and (**b**) is the trajectory from Goal 1 to Goal 2, where the cyan color is the trajectory of global panning from Navigation module and the blue color is the estimated trajectory from the encoder (odometry) of Motion Controller module.

**Figure 13:**
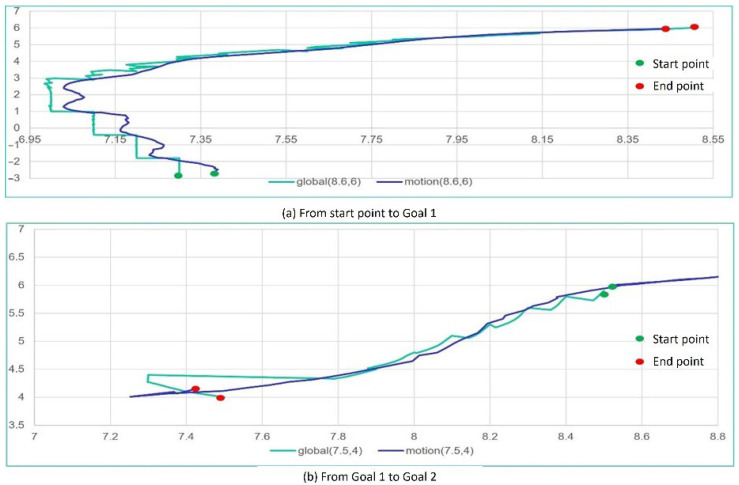
The position trajectory of the tricycle forklift with autonomous navigation in real experiment. (**a**) is the trajectory from start pose to Goal 1, and (**b**) is the trajectory from Goal 1 to Goal 2, where the cyan color is the trajectory of global panning from Navigation module and the blue color is the estimated trajectory from the encoder (odometry) of Motion Controller module.

The movement of the tricycle forklift in the map as shown in Figure 11 includes simulation in the ROS system and a real experiment in exact classrooms. The simulation relies on Gazebo in the ROS system to achieve the tricycle forklift moving in the map of Figure 11. Figure 12 is movement position trajectories under full autonomous navigation. Similarly, Figure 13 is the experiment of the real tricycle forklift. The position trajectories as shown in Figure 12 and Figure 13 are very similar to demonstrate that the model of the tricycle forklift constructed in the ROS system is feasible.

To evaluate the reliability of the ROS navigation, the simulation and real experiment are conducted five times. The results of navigation by Gazebo simulation are shown in Table 4 and Table 5 for arriving at Goal 1 and Goal 2, respectively. Table 6 and Table 7 show the experimental results of the real-world trips to Goal 1 and Goal 2, respectively. The results in the tables are shown by meaningful variables, including mean, deviation, RMSE (Root Mean Square Error), and 95% CI (Confidence Interval). The results of both simulation and real experiments reveal that the tricycle forklift can be navigated and controlled well in a warehouse. However, such a pose error makes it hard to lead the fork to plug into in a pallet. In warehouses, other aid devices such as cameras or LiDAR are designed for accuracy. Alperen Keser and Pınar Oğuz Ekim designed Kinect for the action [23].

In experimental results, the tricycle forklift is navigated by better trajectory tracking in the full autonomous function. Because there is a smaller change in angle between the starting pose and the target pose. Large angle changes during movement can lead to deviations in the robot’s positioning and pose, which causes the trajectory tracking error. The tracking error resulted from the latency of ROS. After receiving the sensor signal from LiDAR, the ROS modules in IPC need nearly 50 ms to send a command to the motor controller of the tricycle forklift. The 50 ms delay is the major source of tracking error. In the future, the computation capability of IPC can be upgraded to reduce the tracking error.

## 6. Conclusions and Future Works

In this study, the forward and inverse kinematics of the Motion Controller in ROS are modified for a tricycle forklift. To demonstrate the developed software function, a scaled-down version of a real tricycle forklift is implemented for experiment testing. Therefore, the real tricycle forklift is installed with LiDAR and related sensors to run the ROS system. Both the sensor model and the motion model contribute to AMCL for getting the pose of the tricycle forklift. The pose of the tricycle forklift is provided to the Navigation module for tracking the planning path. Then, the TEB (Timed Elastic Band) algorithm produces the exact command to control the tricycle forklift. Finally, the Motion Controller module developed for a tricycle forklift, as shown in Figure 3, is demonstrated to be compatible with all of the ROS modules. The demonstration is developed by both simulation and practical experiments. In simulation, Gazebo is developed for the control of the tricycle forklift. In the practical experiments, the classrooms are organized for a warehouse scenario for the cruising test of the real tricycle forklift. Both simulation and practical experiments demonstrate that the tricycle forklift can be navigated and controlled under ROS for enough accuracy for warehouse application. The research results show that tricycle forklifts can be run by ROS for warehouse applications.

For further development, the tricycle forklift system will challenge the performance in complex environments such as dynamic obstacle avoidance. This challenge will focus on improving the Navigation module in Figure 1. Generally, the SLAM engaged in this paper is called LiDAR SLAM, because the sensor is LiDAR, as shown in Figure 1. However, the sensor by visual (camera) is a more reasonable SLAM. It will be easier to apply Generative Artificial Intelligence (GAI). Therefore, Visual SLAM will replace LiDAR in the future for the application of popular GAI.

The new version of ROS is ROS2, which provides multiple masters among connection nodes. However, their communication needs the Data Distribution Service (DDS) protocol of ROS2, which is not totally free. Thanks a lot to the reviewer for reminding us that some DDS protocols are free. Thus, we figure out that DDS protocols of eProsima Fast and Eclipse Cyclone are free. In the future, we will try to apply ROS2 based on the free DDS protocols for easy delivery of products.

Furthermore, tricycle forklifts are very popular in industrial warehouses. This study paves the way to reduce the constructing cost of industrial warehouses. However, the limitation of the tricycle forklift developed in this paper will be studied in the future. For example, the pose error at a goal location is not enough to let the fork plug in a pallet. We will design cameras for the plugging action in the future.

## Figures and Tables

**Table 1:** Environment parameters.

Variable	d_1_	d_2_	d_3_	d_4_	d_5_	d_6_
Length (m)	5.2	7.2	0.8	2.7	6.1	7.5

**Table 2:** The parameters of the tricycle forklift implemented for this study.

Variable	$D_{1}$	$D_{2}$	$D_{3}$	$D_{4}$	$D_{5}$
Length (mm)	765	315	125	1220	490
**Variable**	$D_{6}$	$D_{7}$	$D_{8}$	$D_{9}$	
Length (mm)	160	465	280	75	

**Table 3:** The error between real world and Gmapping.

Variables	Length of Real World (m)	Length of Map (m)	Error (m)
d1	5.2	5	0.2
d2	7.2	7.1	0.1
d3	0.8	0.7	0.1
d4	2.7	2.7	0
d5	6.1	6.1	0
d6	7.5	7.5	0

**Table 4:** The pose error of planned and navigation for moving to Goal 1 in Gazebo.

Pose Error	Mean	Deviation	RMSE	95% CI for Mean Error
x (m)	−0.060	0.127	0.129	[−0.2180, 0.0980]
y (m)	−0.004	0.081	0.073	[−0.1047, 0.0967]
θ (rad)	−0.006	0.071	0.064	[−0.0938, 0.0826]

**Table 5:** The pose error of planned and navigation for moving to Goal 2 in Gazebo.

Pose Error	Mean	Deviation	RMSE	95% CI for Mean Error
x (m)	−0.050	0.073	0.082	[−0.1404, 0.0404]
y (m)	0.046	0.090	0.092	[−0.0653, 0.1573]
θ (rad)	0.007	0.197	0.176	[−0.2370, 0.2510]

**Table 6:** The pose error of planned and navigation for moving to Goal 1 in experiments.

Pose Error	Mean	Deviation	RMSE	95% CI for Mean Error
x (m)	0.036	0.100	0.097	[−0.0884, 0.1604]
y (m)	0.054	0.074	0.085	[−0.0375, 0.1455]
θ (rad)	0.023	0.048	0.048	[−0.0362, 0.0818]

**Table 7:** The pose error of planned and navigation for moving to Goal 2 in experiments.

Pose Error	Mean	Deviation	RMSE	95% CI for Mean Error
x (m)	0.030	0.581	0.521	[−0.6914, 0.7514]
y (m)	0.056	0.173	0.165	[−0.1592, 0.2712]
θ (rad)	−0.080	0.248	0.236	[−0.3878, 0.2278]

## Data Availability

The data presented in this study are available on request from the corresponding author.

## References

[1] Tang H., Cheng X., Jiang W., Chen S. (2021). Research on equipment configuration optimization of AGV unmanned warehouse. IEEE Access.

[2] Guizzo E. (2008). Three engineers hundreds of robots one warehouse. IEEE Spectr..

[3] Ackerman E. (2022). A Robot for the Worst Job in the Warehouse: Boston Dynamics’ Stretch can move 800 heavy boxes per hour. IEEE Spectr..

[4] Le-Anh T., De Koster M.B.M. (2006). A review of design and control of automated guided vehicle systems. Eur. J. Oper. Res..

[5] Alatise M.B., Gerhard P. (2020). Hancke. A review on challenges of autonomous mobile robot and sensor fusion methods. IEEE Access.

[6] Keser A., Ekim P.O. Otonom Forklift ile Palet Tespiti ve Hassas Yanaşma Pallet Detection and Docking with Autonomous Forklift. Proceedings of the 2024 Innovations in Intelligent Systems and Applications Conference (ASYU).

[7] Robot Operating System (ROS). https://www.ros.org/.

[8] Sardinha E.N., Sarajchi M., Xu K., Pineda M.I., Cifuentes C., Munera M. Real-Time Feedback on Older Adults Exercise: A Socially Assistive Robot Coaching System. Proceedings of the 2024 IEEE-RAS 23rd International Conference on Humanoid Robots (Humanoids).

[9] Balasuriya B.L.E.A., Chathuranga B.A.H., Jayasundara B.H.M.D., Napagoda N.R.A.C., Kumarawadu S.P., Chandima D.P., Jayasekara A.G.B.P. (2016). Outdoor robot navigation using Gmapping based SLAM algorithm. Proceedings of the 2016 Moratuwa Engineering Research Conference (MERCon).

[10] Harik E.H.C., Korsaeth A. (2018). Combining hector slam and artificial potential field for autonomous navigation inside a greenhouse. Robotics.

[11] Zhang X., Lu G., Fu G., Xu D., Liang S. (2019). SLAM algorithm analysis of mobile robot based on lidar. Proceedings of the 2019 Chinese Control Conference (CCC).

[12] Dellaert F., Fox D., Burgard W., Thrun S. (1999). Monte carlo localization for mobile robots. Proceedings of the 1999 IEEE International Conference on Robotics and Automation (Cat. No. 99CH36288C).

[13] Wasisto I., Istiqomah N., Trisnawan I.K.N., Jati A.N. Implementation of Mobile Sensor Navigation System Based on Adaptive Monte Carlo Localization. Proceedings of the 2019 International Conference on Computer, Control, Informatics and Its Applications (IC3INA).

[14] Cui S.-G., Wang H., Yang L. A simulation study of A-star algorithm for robot path planning. Proceedings of the 16th International Conference on Mechatronics Technology.

[15] Wang P., Zhang H., Tang Y. (2025). Path Planning in Narrow Road Scenarios Based on Four-Layer Network Cost Structure Map. Sensors.

[16] Rösmann C., Hoffmann F., Bertram T. (2015). Timed-elastic-bands for time-optimal point-to-point nonlinear model predictive control. Proceedings of the 2015 European Control Conference (ECC).

[17] Wu J., Ma X., Peng T., Wang H. (2021). An Improved Timed Elastic Band (TEB) Algorithm of Autonomous Ground Vehicle (AGV) in Complex Environment. Sensors.

[18] Gazebo. https://gazebosim.org/home.

[19] Navigation (ROS). https://wiki.ros.org/navigation.

[20] Grisetti G., Kümmerle R., Stachniss C., Burgard W. (2010). A tutorial on graph-based SLAM. IEEE Intell. Transp. Syst. Mag..

[21] Aini F.R.Q., Jati A.N., Sunarya U. (2016). A study of Monte Carlo localization on robot operating system. Proceedings of the 2016 International Conference on Information Technology Systems and Innovation (ICITSI).

[22] Li W., Qiu J. (2025). An Improved Autonomous Emergency Braking Algorithm for AGVs: Enhancing Operational Smoothness Through Multi-Stage Deceleration. Sensors.

[23] tf (ROS). https://wiki.ros.org/tf.

[24] Kümmerle R., Grisetti G., Strasdat H., Konolige K., Burgard W. (2011). G^2^o: A general framework for graph optimization. Proceedings of the 2011 IEEE International Conference on Robotics and Automation.

